# [Retracted] Downregulation of Bmi-1 is associated with suppressed tumorigenesis and induced apoptosis in CD44^+^ nasopharyngeal carcinoma cancer stem-like cells

**DOI:** 10.3892/or.2025.9032

**Published:** 2025-12-04

**Authors:** Xinhua Xu, Yang Liu, Jin Su, Daojun Li, Juan Hu, Qiao Huang, Mingqian Lu, Xiaoyan Liu, Jinghua Ren, Weihong Chen, Lidan Sun

Oncol Rep 35: 923–931, 2016; DOI: 10.3892/or.2015.4414

Following the publication of the above article, a concerned reader drew to the Editor's attention that one set of the tumor data comparing between the ‘CD44^+^’ experiments in Fig. 4A and the ‘CON’ experiments in Fig. 4B on p. 428 had apparently been duplicated; moreover, several of the images of various of the mice shown in this figure looked more similar in appearance than might have been expected. Furthermore, upon performing an independent analysis of the data in this paper in the Editorial Office, it came to light that the data panel showing the results of the migratory assay experiment relating to the ‘KD’ group in Fig. 2B on p. 926 contained an internally duplicated area that would have been difficult to attribute to coincidence.

The authors were asked for an explanation to account for these concerns, but the Editorial Office did not receive a reply. Therefore, the Editor of *Oncology Reports* has decided that this paper should be retracted from the Journal on account of a lack of confidence in the presented data. The Editor apologizes to the readership for any inconvenience caused.

